# Editor's Letter-Box

**Published:** 1897-04-10

**Authors:** 


### Editor's Letter-Box.

[Our Correspondents are reminded that prolixity is a great bar to publication, and that brevity of style and conciseness of statement greatly facilitate early insertion.]

Dr. J. Barker Smith writes: The deeply interesting article on the arrest of puerperal fever by inhibition of the pulse, The Hospital, page 413, by Dr. Glynn Whittle, leads me to send a few original notes on the use of aconite. In the first place, aconitine, atropine, cocaine, and hyoscyamine are not immediately negatived by permanganate of potash; in this respect they differ from morphine, apomorphine, veratrine, colchicine, &c. I share the diffidence of Dr. Glynn Whittle in the use of aconite preparations, although I am convinced of their extreme value. I do not know any drug which has given me so much satisfaction in neuralgic pains in alcoholics and others, used as an application. The literature of the subject has convinced me that all preparations of aconite should be carefully standardised organoleptically, physiologically as well as chemically, and that a certificate should accompany all preparations of aconite. Again, there is a marked distinction to be made between aconite preparations and solutions of the alkaloid aconitine; many would forbid the use of the alkaloid in medicine. Amorphous aconitine is as redoubtable as the crystalline as a cause of fatalities, yet the former is negatived by permanganate of potash, while the crystalline aconitine forms stable combinations.

The Monkshood Plant: A plant in flower on the window-sill outside an open window soon produces some evident effects upon the more sensitive occupants of the room. Delicate tinglings of lips and tip of the tongue, and in a day or two lassitude. So in the surgery; however carefully we measure or apply aconite preparations, we shall be reminded of such use by these subsequent tinglings. Such I consider a test of very great forensic importance; let us try and appreciate the quantities which produce such effects. Some people are more sensitive than others, such will detect aconitine tinglings from so small a quantity as a cubic centimètre of one per million solution held for one minute in the mouth, a few minutes afterwards the tinglings are detected. Most people will detect one per hundred thousand solution; now, such a quantity is only one hundredth of a milligram of alkaloid, or one six-thousandth of a grain. In other words, a medicinal dose of crystallised aconitine will afford a hundred sensory experiments, accompanied or followed by cardiac depressions. In other words, we may by distributed doses, with one maximum dose of tincture of aconite, keep up the prophylactic effects for the whole month of the puerperium, or during a month's exposure in a plague or fever-infested district. A square centimètre of the corolla itself will give four or more sensory experiments; the nectar contained ten per cent. glucose, and appeared free from alkaloid. About nineteen species of monkshood are mentioned by Philip Miller, F.R.S., Gardener to the Worshipful Company of Apothecaries at their Botanic Garden in Chelsea.—"The Gardener's Dictionary," 1748. This authority says of one species, aconitum salutiferum, "the wholesome wolf's-bane, used in physic, and is supposed to be an antidote to the poison of wolf's-bane," *i.e.*, to monkshood or aconite. We may ask with respect to this statement two important questions. Are our preparations of aconite dangerously variable as regards alkaloid percentage. Pharmaceutical chemistry answers this question somewhat in the negative. The next question is whether we have not in the other constituents of the aconite plant, when the alkaloid is removed, modifiers of the physiological action of the aconitine; in other words, an antidote to aconitine. Pharmacy and Therapy. So much has the importance of accuracy in dosage impressed me that my note book contains the following calculation for my own guidance: "A milligram of aconitine is four large doses, and we are not justified in giving more. Such a milligram of aconitine may be expected in eight minims of aconite liniment or in forty minims of aconite tincture. Never use externally in painting the skin more than ten minims of aconite liniment, nor is it wise to ever prescribe more in any liniment entrusted to the patient, *e.g.*, linim. aconite, ℳx.; chloroform, ʒj.; linim. saponis, ad. ℥j." I believe it was John Hunter who said, "Give me a constrictor of the vessels, and I will cure all inflammations." We have such in ergot, aconite, digitalis, &c., and apparently we are demonstrating the truth of such aphorism. We all know pain, once started, goes on by compound interest, and aconite is one of the best analgesics for a topical remedy. Its best effects are not immediate, but remote; the lethargic tongue of the experimenter is felt the day after his experiments with aconite preparations. Certain it is that such applications are not symptom remedies, but causal and permanent. The *primâ facie* evidence of integrity of aconite preparations I consider rests in a definite dilution and test of tingling, and slowing of heart; colour, reduction of Fehling's solution, expression of oxidation capacity with acid permanganate, and turbidity with iodine. Indeed, organoleptic, physiological, and chemical tests.

